# Tissue clearing applications in memory engram research

**DOI:** 10.3389/fnbeh.2023.1181818

**Published:** 2023-08-28

**Authors:** Kwok Yui Tony Yip, Johannes Gräff

**Affiliations:** Laboratory of Neuroepigenetics, Brain Mind Institute, School of Life Sciences, École Polytechnique Fédérale de Lausanne (EPFL), Lausanne, Switzerland

**Keywords:** memory engram, tissue clearing, neuronal circuit, neuroepigenetics, immunolabeling-enabled three-dimensional imaging of solvent-cleared organs, engram complex

## Abstract

A memory engram is thought to be the physical substrate of the memory trace within the brain, which is generally depicted as a neuronal ensemble activated by learning to fire together during encoding and retrieval. It has been postulated that engram cell ensembles are functionally interconnected across multiple brain regions to store a single memory as an “engram complex”, but visualizing this engram complex across the whole brain has for long been hindered by technical limitations. With the recent development of tissue clearing techniques, advanced light-sheet microscopy, and automated 3D image analysis, it has now become possible to generate a brain-wide map of engram cells and thereby to visualize the “engram complex”. In this review, we first provide a comprehensive summary of brain-wide engram mapping studies to date. We then compile a guide on implementing the optimal tissue clearing technique for engram tagging approaches, paying particular attention to visualize engram reactivation as a critical mnemonic property, for which whole-brain multiplexed immunostaining becomes a challenging prerequisite. Finally, we highlight the potential of tissue clearing to simultaneously shed light on both the circuit connectivity and molecular underpinnings of engram cells in a single snapshot. In doing so, novel brain regions and circuits can be identified for subsequent functional manipulation, thus providing an opportunity to robustly examine the “engram complex” underlying memory storage.

## The emerging concept of an “engram complex”

A memory engram is defined as the physical substrate of the memory trace within the brain (Josselyn et al., 2015; Josselyn and Tonegawa, 2020). The term “engram” was first introduced by the German Biologist Richard Semon (Semon, 1904), who postulated that memory encoding activates a population of neurons that undergo off-line, persistent chemical and/or physical changes to form and store acquired information as an engram. Subsequent reactivation of the engram by retrieval cues would then induce memory recall (Semon, 1921, 1923) (Box 1). For the past decade, modern engram tagging techniques that allow for the visualization, manipulation, and isolation of neuronal ensembles at single-cell resolution have successfully transformed Semon's pure theoretical proposal into concrete experimental frameworks.

Box 1 **Defining the engram.**

The term “engram” was introduced by Semon more than 100 years ago as the physical substrate of the memory trace within the brain (Semon, 1904). Semon defined that an engram should satisfy four core criteria: Persistence, ecphory, content, and dormancy. First, an engram is a persistent change in the brain, resulting from a specific experience. Second, an engram is subjected to ecphory, meaning that an engram can be behaviorally reactivated. Third, an engram is content-specific to faithfully recall what has been encoded. Fourth, an engram may exist in a dormant state during the offline period between encoding and retrieval. Through contemporary research efforts, these defining criteria have now become prevalent and substantiated by biological evidence (Josselyn et al., 2015; Albo and Gräff, 2019; Josselyn and Tonegawa, 2020).

Visualizing or “tagging” the engram relies on immediate-early gene (IEG) expression such as *cFos, Arc*, or *Zif268*, which marks neurons with heightened neuronal activity (Box 2)—for instance, *cFos* expression induced by a learning event (Guzowski et al., 2005; Stone et al., 2011). Coupled with reporter genes under the influence of inducible IEG promoters, cells activated by memory encoding can thus be tagged with

Box 2 ***cFos* as a neuronal activity and putative engram marker.**

Over the years, *cFos* together with several other IEGs has been widely used as a marker of the activated neuronal ensemble during behavior (Sheng and Greenberg, 1990; Yap and Greenberg, 2018) Shown in the striatum. In the striatum and hippocampus of intact behaving rats or mice, *cFos* expression is mediated by the neuronal activation of the ERK/MAPK pathway. High levels of calcium influx through NMDA receptors and voltage-sensitive calcium channels activate ERK/MAPK-dependent phosphorylation of ELK1-SRF and hence ribosomal S6 kinase (RSK)-dependent phosphorylation of CREB, which in turn binds to the promoter region of *cFos*. Recently, Yap et al. (2020) demonstrated that glutamatergic excitatory input along with bidirectional modulation by GABA inhibitory input plays a major role in shaping the *cFos*-induced neuronal ensemble. cFos protein as the product then interacts with other IEG products, e.g., cJun to form a heterodimeric transcriptional factor complex called AP-1 to activate downstream targets including *jdp2* (Fernandez-Albert et al., 2019) and *Gabrb3* (Marco et al., 2020). A detailed summary of the molecular and cellular mechanisms of *cFos* as the marker of neuronal activity during behavior can be found in Cruz et al. (2013).

Putting back in the context of the memory engram research, *cFos* alone must be seen as a marker of a *putative* memory engram, as *cFos* only labels the learning-activated neuronal ensemble, agnostic to their possible reactivation at later times. It is only with the use of engram tagging techniques that reactivation can be measured and thus a once *cFos*-positive cell be qualified as truly belonging to the engram. The latest study by Jung et al. (2023) illustrated that using multiple behavior tasks, retrieval cues that best overlap with training cues produce maximal memory recall via maximal engram reactivation. This provides strong evidence to link reactivated engram neurons with memory performance. Finally, selective manipulation of reactivated engram neurons can further validate this link, which can be accomplished with, e.g., a *cFos* tTA-tet tag and lentivirus-based genetic targeting system in combination with a tamoxifen-inducible Cre-loxP recombination system (Yokose et al., 2017).

an enduring label to mimic the endogenous IEG. When the tagging window is closed to switch off reporter gene expression, cells activated by memory recall can be labeled by endogenous IEG activity at later times. With immunohistochemistry (IHC) to show cellular colocalization between the IEG-tagged reporter and the IEG itself, engram reactivation can be measured as the overlap between the encoding ensemble and the recall ensemble, one of the four core criteria for a cell to be qualified as an engram cell (Josselyn et al., 2015) (Box 2).

There are different tagging techniques, e.g., TetTag (Reijmers, 2007), TRAP1 (Guenthner et al., 2013), TRAP2 (DeNardo et al., 2019), and ArcERT2 (Denny et al., 2014) reporter mouse lines or viral approaches (Hsiang et al., 2014; Ryan et al., 2015) to identify engram cell ensembles for multiple memory tasks including contextual fear conditioning (Liu et al., 2012), auditory fear conditioning (Reijmers, 2007), reward learning (Redondo et al., 2014), and drug addiction (Hsiang et al., 2014). Most of these observational engram studies have revealed that the engram reactivation rate is approximately 10–15%.

Most engram studies have examined only one specific brain region at a time, which includes different hippocampal subregions including CA1 (Sekeres et al., 2010), CA3 (Choi et al., 2018), and the DG (Sekeres et al., 2010, 2012; Liu et al., 2012; Park et al., 2016), different parts of the amygdala including LA and BLA (Redondo et al., 2014; Ryan et al., 2015) and cortical areas such as the medial prefrontal (Yang et al., 2016; Kitamura et al., 2017; Abdou et al., 2018) or retrosplenial cortex (Cowansage et al., 2014). Nevertheless, Semon's engram theory states a “law of engraphy” in which “all simultaneous excitations … form a connected simultaneous complex of excitations which, as such, act engraphically, that is to say leaves behind it a connected, and to that extent, unified engram complex” (Semon, 1923). And even beyond the field of engram research, it is well established that memory storage is not confined to a single-cell ensemble localized in a single brain site, but is rather represented by a network of such ensembles distributed and connected in dispersed brain regions (Tonegawa et al., 2015; Tovote et al., 2015). As such, a holistic brain-wide engram mapping is undoubtedly a prerequisite for putting Semon's “engram complex” hypothesis to test even though several functional engram studies using chemogenetic, optogenetic, or pharmacological tools (Cruz et al., 2013) have laid the foundation of testing this hypothesis (Han et al., 2009; Liu et al., 2012; Cowansage et al., 2014) by targeting specific interregional connections, mainly hippocampal-cortical projections for contextual fear memory.

## Conventional attempts for brain-wide engram mapping

Brain-wide attempts to map neuronal ensembles activated by memory have only started to surface over the past decade. In one of the first studies, Wheeler et al. (2013) performed conventional IHC of *cFos-*stained brain slices and thereby generated a near brain-wide activation map between 1-day, i.e., recent recall vs. 36-day, i.e., remote recall of a contextual fear memory. Regional *cFos* quantification across 84 brain regions identified active brain regions; interregional correlation analysis identified a collection of brain regions where *cFos* expression co-varied across mice; and graph theory deconstructed the functional connectivity underlying memory recall. Although long been thought so (Wheeler et al., 2013; Tonegawa et al., 2015; Tovote et al., 2015), these results are among the first to elucidate that long-term fear memory is an emergent property of a functional brain network, which manifests small-world properties—allowing for local information processing in more densely connected clusters (Bullmore and Sporns, 2009)—with a subset of highly interconnected “hub” nodes including the anterior cingulate cortex (ACC), prelimbic (PL) cortex, and thalamic nucleus reuniens (NRe), which potentially play privileged roles in memory expression. This fear memory network is featured within a hippocampal–thalamic–cortical core, but from recent memory to remote memory, there is a mnemonic shift of functional connectivity from the dorsal hippocampus to distributed neocortex. Thus, the Wheeler et al. (2013) study not only significantly expanded earlier reports highlighting a spatiotemporal shift during remote memory consolidation (Frankland and Bontempi, 2005), but also pinpointed hubs that had previously been missed by targeted region-specific approaches.

Based on Wheeler et al. (2013)'s correlational hypothesis, Vetere et al. (2017) confirmed the causal necessity of these network nodes via post-training chemogenetic silencing of 21 out of the 84 brain regions. Silencing highly connected network hubs, e.g., dorsal CA1, laterodorsal thalamic nucleus, lateral septum, and NRe, produced the largest memory impairment upon memory recall 10 days later. Based on these grounds, these hubs in subsequent studies have been more thoroughly investigated and hitherto unexpected roles discovered, like an eminent role for the NRe in recent and remote fear memory extinction (Ramanathan et al., 2018a; Silva et al., 2018, 2021; Ramanathan and Maren, 2019).

Together, these two studies demonstrate that brain-wide mapping and functional manipulations using traditional methodologies can provide holistic perspectives on network connectivity and define novel targets for subsequent in-depth manipulations. Nevertheless, these studies are considered “brain-wide activity mapping”, rather than engram-specific approaches, as *cFos* activation alone does not satisfy the necessary criteria of engram reactivation (Boxes 1, 2). Another technical limitation comes from conventional brain slice IHC, which is labor-intensive and prone to signal loss due to mechanical sectioning. Owing to these technological barriers, true brain-wide engram mapping had yet to see the light of day.

## Tissue clearing for whole-brain 3D imaging

Standard histological methods have been revolutionized by the rapid development of tissue clearing within the past decade, which renders the biological tissue transparent to minimize light scattering (Ueda et al., 2020). Optical clearing chemistry is accompanied by whole-tissue staining to label deeply across thick tissue (Marx, 2016), while conventional mechanical sectioning is replaced by advanced optical sectioning with light-sheet microscopy, which illuminates the transparent specimen with a thin sheet of lateral laser light for image acquisition (Reynaud et al., 2015). Furthermore, light-sheet microscopy allows for high-speed whole-tissue 3D imaging at nearly isotropic single-cell resolution (Franceschini et al., 2020) and can thereby rapidly generate large multidimensional image data. The integrated workflow of brain atlas registration, brain region segmentation, and automated cell counting is constantly being updated by the invention of more accurate, realistic, and efficient computational pipelines (Long et al., 2012; Amat et al., 2015).

In neuroscience, whole-brain 3D imaging not only circumvents the technical obstacle of laborious mechanical sectioning, which may turn impractical and prone to information loss for brain-wide mapping studies, but also pushes hitherto non-addressable questions within experimental reach through the view of global patterning. Therefore, the recent explosive proliferation of optical clearing protocols is not surprising.

Spanning the wide variety of tissue-clearing protocols, the fundamental principle to achieve optical transparency is to extract water and lipid from the tissue as the primary source of light scattering, homogenize the refractive index (RI) within the tissue, and immerse it in a RI matching solution so that light scattering is minimized (Spalteholz, 1914). Theoretically, a perfect RI matching between cleared tissue and the solution would yield perfect transparency, regardless of any RI values. The cleared tissue RI depends mainly on how strong the dehydration and delipidation are designed and implemented in the protocol. Thus, this creates a discrepancy and practically determines the outcome of the transparency degree. Nowadays, there are three major categories of tissue-clearing protocols: hydrogel-based clearing methods, hydrophilic/aqueous-based, and hydrophobic/solvent-based.

The hydrogel-based clearing methods, e.g., CLARITY (Chung et al., 2013), use an acrylamide monomer to form a tissue-hydrogel hybrid, which anchors the biomolecules by cross-linking. After forming the hydrogel, the sample is then delipidated with sodium dodecyl sulfate (SDS) detergent. CLARITY is particularly effective in preserving endogenous fluorescence for imaging. The entire clearing protocol of multiple weeks can be accelerated with electrophoresis, but then needs special equipment to assist in clearing to achieve tissue transparency (RI~1.40–1.50). One unique feature of hydrogel-based clearing is the inherent possibility for tissue expansion, which subsequently span off into the development of expansion microscopy (ExM) for super-resolution microscopy (Chen et al., 2015). We guide the reader to review the potential application of ExM in neuroscience and light-sheet imaging to Parra-Damas and Saura (2020) and Daetwyler and Fiolka (2023), respectively. Commercial equipment is readily available for the routine application of CLARITY, but relatively rare for the vast variants of CLARITY-based protocols for tailor-made use. For example, SHIELD-eFLASH enhances biomolecule preservation to better endure whole-mount labeling via novel epoxide linkers (Park et al., 2018), drives antibody penetration via stochastic electrotransport and improves antibody binding affinity by altering the pH and detergent conditions (Yun et al., 2019). A detailed review of hydrogel-based clearing can be found in Gradinaru et al. (2018).

Hydrophilic/aqueous-based clearing methods, e.g., CUBIC (Susaki et al., 2015), use various water-soluble reagents such as sugars, dextran, sucrose, urea, and amino alcohols, to passively homogenize the RI throughout the tissue. Using aqueous reagents is easier to implement due to higher biocompatibility and biosafety, and can achieve satisfactory transparency (RI~1.37–1.52) within a week. With delipidation and hyperhydration agents, clearing can be further accelerated plus mild tissue expansion can be obtained to facilitate whole-tissue labeling. A detailed review of hydrophilic/aqueous-based clearing can be found in the study by Tainaka et al. (2018).

Hydrophobic/solvent-based clearing methods, e.g., 3DISCO (Ertürk et al., 2012), use a cocktail of organic solvents to perform dehydration, delipidation, and RI matching. By replacing water and lipids as the main source of light scattering and then homogenizing the RI between the remaining tissue scaffold and the organic RI matching solution, full tissue transparency (RI~1.52–1.56) can be achieved within 2–3 days. The resulting solid tissue “crystal” facilitates mounting to the microscope. On the flipside, the vigorous usage of organic solvents often causes tissue shrinkage plus quenching of endogenous fluorescence. These disadvantages have been leveraged and circumvented by variants of the original DISCO protocols. iDISCO+ (Renier et al., 2016) utilizes a gradient of methanol/water mixture for dehydration, dichloromethane for delipidation, and dibenzyl ether for RI matching. iDISCO+ not only maintains normal brain size to facilitate brain atlas registration, but also maximizes the compatibility to whole-mount immunostaining via enhanced antibody penetration with stronger delipidation and dehydration. Quenched endogenous fluorescent proteins, e.g., GFP/RFP can instead be visualized with anti-GFP/RFP antibodies. A modified version of iDISCO+ called AdipoClear (Friedmann et al., 2020) further improves delipidation and hence lowers tissue autofluorescence. With the latest development of Fast3DClear (Kosmidis et al., 2021) and EZClear (Hsu et al., 2022), which incorporates aqueous-based RI matching into solvent-based dehydration and delipidation, endogenous fluorescence can be preserved while performing whole-mount immunofluorescence simultaneously. In addition, a preprint from Kanatani et al. (2022) invented DISCO clearing-based whole-brain fluorescence *in situ* hybridization (FISH) called TRIC-DISCO for labeling RNA as well. One unique feature of DISCO-based clearing is the ease of reverse clearing which restores the transparent brain back to the opaque form for aqueous processing, e.g., traditional IHC. A detailed review of hydrophobic/solvent-based clearing can be found in Molbay et al. (2021).

Tissue clearing permits previously unseen, global visualization of 3D images over a whole tissue or body, which can potentially unveil unknown, inaccessible structural details of neural architectures. This is particularly crucial both in (1) dissecting continuous anatomical structures and (2) sampling very sparse targets over large volumes of tissue. In neuroscience too, brain clearing has been most commonly used in 3D axonal projectome mapping (Ren et al., 2018; Friedmann et al., 2020; Liu et al., 2022) and brain-wide engram mapping (Renier et al., 2016; Schneeberger et al., 2019), the latter of which will be outlined in the next section.

## Tissue clearing for brain-wide engram mapping

In simple terms, brain-wide engram mapping is the technically advanced version of brain-wide activity mapping, using genetically engineered activity tagging systems. While activity mapping has been restricted to only one timepoint per animal in the course of a given phenotype, engram mapping can reliably reveal two distinct timepoints of the same behavior, by merging whole-brain labeling of the IEG-driven reporter protein with the endogenously triggered IEG itself. The rapid and almost simultaneous advancement of both engram tagging and optical clearing technologies, therefore, presents a golden opportunity to launch comprehensive brain-wide engram mapping.

Nevertheless, brain-wide engram mapping is far from being a standard procedure as the optimization obstacles regarding optical clearing are still a major concern and require tremendous trial-and-error efforts. For example, Pavlova et al. (2018) used ArcCreERT2 × ChR2-eYFP mice to study contextual fear memory engrams in a brain-wide manner. They tested all leading clearing protocols at the time to find the ideal compatibility of whole-brain co-labeling and engram tagging combined with *cFos* or *Arc* IHC. CLARITY greatly distorted the tissue size thus hindering brain atlas registration. Furthermore, CLARITY prevented deep brain *Arc* immunostaining and satisfactory preservation of the endogenous fluorescence emitted by eYFP. iDISCO, in contrast, quenched endogenous eYFP completely but allowed for uniform *Arc* immunostaining. Yet, when co-stained, both the anti-GFP and anti-*Arc* antibodies showed decreased penetration into the brain, which was apparent by a peripheral fluorescent halo around the brain. This decreased penetration was caused by the dense and diffused dendritic labeling of Arc-eYFP, and could not be alleviated by the use of an anti-GFP nanobody, or by non–methanol-based dehydration steps. Finally, a modified CUBIC protocol allowed for endogenous eYFP preservation, both concomitantly with *cFos* or *Arc* immunofluorescence, and a subsequent study later found that iDISCO+ too allowed for anti-eYFP immunostaining. Yet for both protocols, the antibodies still only penetrated in trimmed mm-thick brain sections, not across the whole brain (Leal Santos et al., 2021, 2022).

**Figure 1:**
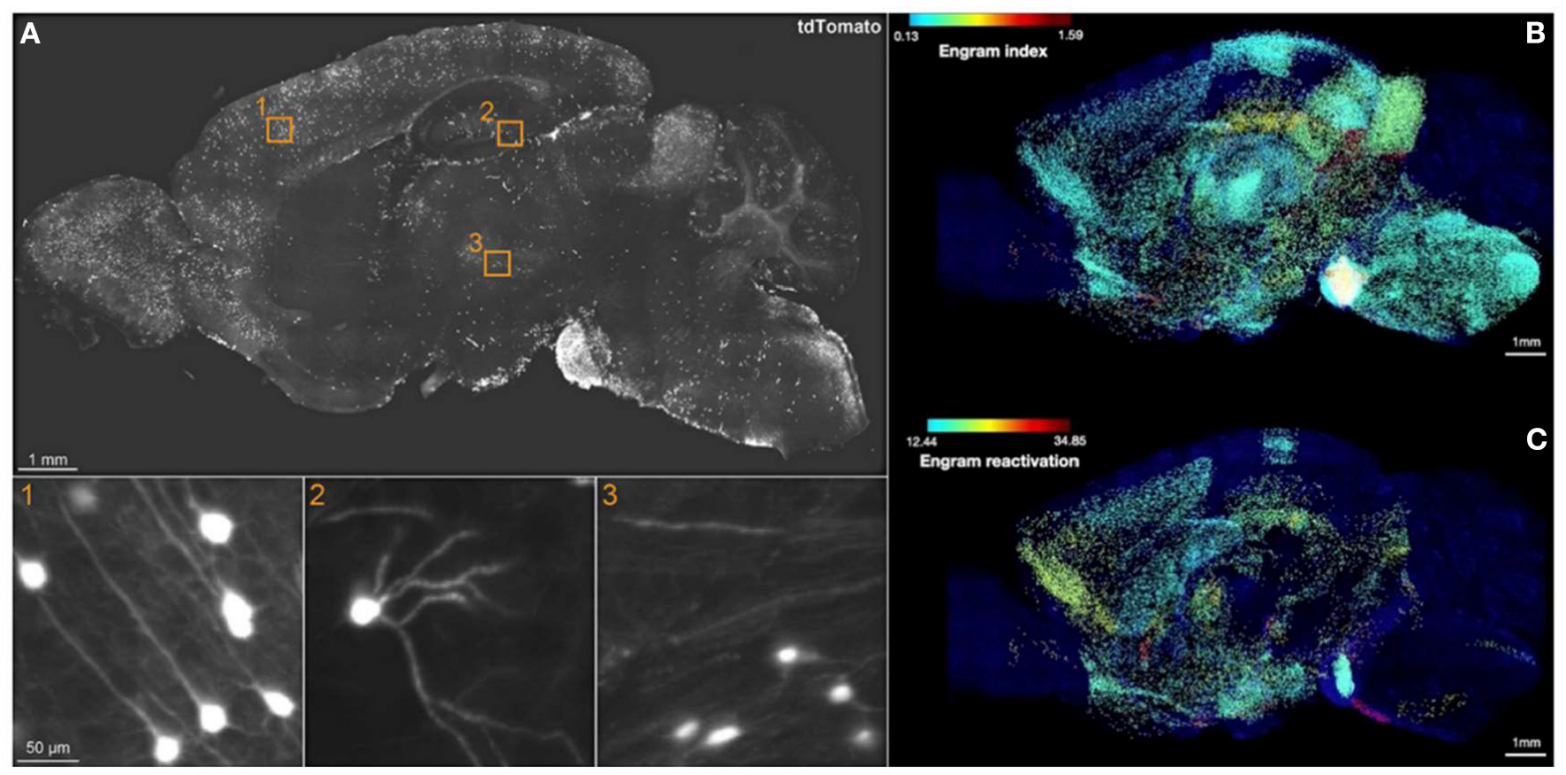
Brain-wide engram map. **(A)** Sagittal view of an optical section from a 3D image of CFC-TRAPed brain, showing tdTomato+ cells in 1) cortex, 2) DG, and 3) thalamus. **(B)** Engram index map and **(C)** engram reactivation rate map. The engram index points to regional coactivation, whereas engram reactivation points to cellular colocalization. Adapted from Roy et al. (2022).

The breakthrough of SHIELD (Park et al., 2018) combined with eFLASH (Yun et al., 2019) techniques, which succeeds in preserving endogenous fluorescence followed by *cFos* immunostaining across a whole hemisphere, has yielded the most comprehensive brain-wide engram mapping thus far. Roy et al. (2022) used the TRAP1 (Guenthner et al., 2013) × tdTomato engram mouse line to mark active neurons, temporally controlled by 4-hydroxytamoxifen (4-OHT) injection. Three behavior cohorts were prepared: a home cage (HC), contextual fear conditioning (CFC), and recent recall group (Re). First, with the brain-wide activity maps of TRAP1-tdTomato+ cells only, the authors derived a so-called engram index by screening brain regions, which are co-active at both encoding and recall. This engram index thus served as the basis of putative engram regions. Next, the authors generated the hitherto first brain-wide colocalization map between TRAP1-tdTomato+ cells upon encoding and *cFos*+ cells upon a 3-day recent recall to fulfill the necessary condition of engram reactivation (Figure 1). This landmark brain-wide “engram reactivation” map pinpointed several novel engram regions. Approximately 60% of 247 analyzed brain regions showed consistent results between the “engram index” and engram reactivation. Furthermore, simultaneous chemogenetic reactivation of engram ensembles in the CA1, BLA, and anteromedial thalamus produced a similar freezing response as induced by natural recall cues, which was not observed when either of these regions was activated alone. This brain-wide engram map thus delineated novel engram regions for functional interrogation to dissect the distributed subsets of the overall memory storage network, and therefore provided the first experimental evidence of the “engram complex”.

Nevertheless, the TRAP1 line has its own caveats, namely the disruption of endogenous *cFos* expression and inefficient accessibility to subcortical regions. DeNardo et al. (2019) thus engineered the TRAP2 line, which exhibits broader genetic access across the whole brain, and preservation of endogenous *cFos*. TRAP2-tdTomato mice also show better compatibility with iDISCO+ clearing owing to decreased dendritic labeling and hence increased antibody penetration. With brain-wide activity mapping of TRAP2-tdTomato+ cells alone, the authors first revealed the activation pattern of a novel environment against home cage exposure, auditory cued fear conditioning against tone-only control, and 1-day recent recall against 14-day remote recall. Based on these maps, the prelimbic (PL) cortex emerged as a highly interconnected hub: Cued fear memory was more accompanied by reactivation of the PL engram at remote than recent times, and this temporal evolution required the initiation of the PL engram at encoding. During recent recall, the PL functionally connected with the hippocampus and central amygdala; but during remote recall, the PL engram reorganized to functionally connect with cortical association areas instead. Finally, the authors compared the whole-brain projection map vs. *cFos* activity map upon PL engram photostimulation to investigate the circuit mechanism. Despite a similar broad structural projectome, the PL engram manifested a shift in the functional recruitment from subcortical regions for recent memory to the neocortex for remote memory. In summary, DeNardo et al. (2019) provided a holistic view of the spatiotemporal shift of distributed circuit connectivity throughout system consolidation using the most updated methodology of brain-wide activity-projectome mapping thus far.

The TRAP2 line has since been widely used for brain-wide activity-projectome mapping of diverse behaviors (Allen et al., 2019; Osterhout et al., 2022; Yang et al., 2023). Nevertheless, it is important to note that there could still be a regional discrepancy in tagging efficacy even when examining the same behavioral time point (Cho et al., 2017; Bonapersona et al., 2022). In other words, there are brain regions where TRAP2 tagging does not fully recapitulate the endogenous cfos expression for as of yet unknown reasons, which can nevertheless potentially be compensated for with virus-based tagging.

### Which clearing protocol should one choose for brain-wide engram mapping?

**Figure 2:**
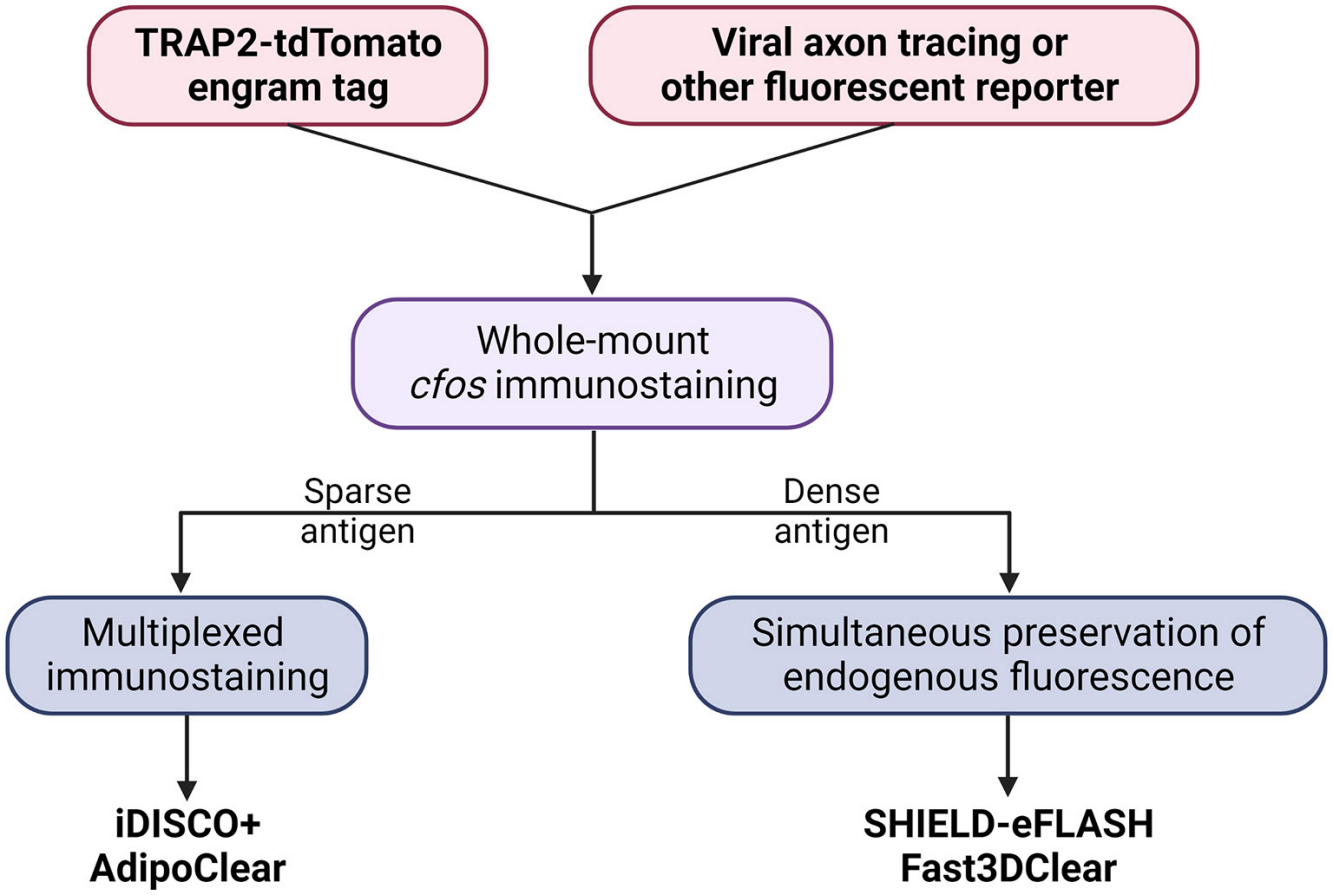
Decision tree to choose the optimal tissue clearing protocol for brain-wide engram mapping.

Here, we present a flowchart to help weigh different parameters and choose the optimal clearing protocol for engram mapping based on our own experience (Figure 2 and Table 1).

**Table 1:** Key features of the clearing protocols selected in Figure 2.

**Protocol**	**Tissue permeabilization method**	**Endogenous fluorescence preservation**	**Whole-mount immunostaining**	**RI matching**	Protocol time^*^	Clearing time^*^	**Tissue size change**	**Application in brain-wide engram mapping**
*iDISCO+* (Renier et al., 2016)	Methanol, DCM, H_2_O_2_ for bleaching	No	Yes; multiplexing	DBE; RI = 1.56	~1 month	2–3 days	Minimal	DeNardo et al., 2019
*AdipoClear* (Friedmann et al., 2020)	Methanol, DCM, B1n buffer at 4°C	No	Yes; multiplexing	DBE; RI = 1.56	~1 month	2–3 days	Minimal	No
*Fast3DClear* (Kosmidis et al., 2021)	50–90% THF, pH9 at 4°C	Yes; preserved best at 4°C	Yes	Iohexol-based solution, then Cargille oil; RI = 1.51–1.54	< 1 month	2–3 days	Slightly expand	No
*SHIELD-eFLASH* (Park et al., 2018; Yun et al., 2019)	Polyepoxide as cross-linker, 300 mM SDS	Yes; preserved best at 45°C	Yes; requires stochastic electrotransport	Iohexol-based PROTOS solution; RI = 1.46	~1 month	~2 weeks	Minimal	Roy et al., 2022

^*^for whole mouse brain hemisphere. DCM, dichloromethane; DBE, dibenzyl ether; RI, refractive index; SDS, sodium dodecyl sulfate; THF, tetrahydrofuran.

Tailor-made for the purpose of brain-wide mapping, TRAP2-tdTomato is currently the most suitable engram tagging mouse line. Whole-brain co-labeling is the necessary prerequisite of cellular colocalization, i.e., engram reactivation. SHIELD-eFLASH or Fast3DClear allows for the preservation of endogenous fluorescence, which best performs together with viral axon tracing to dissect engram cell-specific circuit connectivity. iDISCO+ or AdipoClear, in contrast, allows for whole-brain multiplexed immunostaining which is best suited for dissecting engram cell-specific molecularmechanisms (Boxes 3, 4).

Box 3 **Tips for whole-mount immunostaining.**

Whole-mount immunostaining is mandatory for brain-wide engram mapping but is substantially more challenging than conventional slice IHC. The culprit is the antibody penetration across thick tissue, which is exponentially exacerbated upon co-staining. Conversely, in combination with endogenous fluorescence, there are no perfect win-win scenarios between enhancing antibody penetration and avoiding endogenous fluorescence quenching, regardless of the clearing protocol. Considering the ease and range of applicability, the leverage is therefore driven toward iDISCO+ clearing, which best adapts to whole-brain multiplexed immunostaining by minimizing steric hindrance against antibody infiltration. Nevertheless, it is inevitably the most challenging endeavor. Here, we provide a list of recommendations about whole-mount immunostaining in DISCO clearing based on our own experience (Yip and Gräff, in preparation). A comprehensive tutorial on the technical considerations to establish a successful tissue clearing and imaging experiment can be found in Weiss et al. (2021).

1. Tissue permeabilization is the first concern. Alternative detergents e.g., CHAPS (Frankowski et al., 2022) and β-cyclodextrin (Branch et al., 2021; Mai et al., 2023) drastically facilitate deeper immunostaining. Whether each antibody is compatible with a new chemical has to be carefully tested with brain slice IHC.
2. Whole hemisphere is preferred over whole brain approaches, particularly in co-staining (Frankowski et al., 2022; Roy et al., 2022). This fundamentally reduces physical distance for antibody penetration.
3. A peripheral fluorescent halo is a clear indicator of poor antibody penetration. If staining is still detectable inside the tissue, antibody concentration can be reduced.
4. Each antibody should be incubated for at least 4–5 days. Incubation time and antibody concentration should be increased when an intensity gradient without deep staining and a peripheral halo is observed.
5. Screening a wide variety of antibody strains, especially different hosts and monoclonal antibodies, is time-consuming and expansive, but worth investing. Testing directly in whole tissue is the only way to optimize antibody penetration.
6. For multiplexed immunostaining, sequentially stain from the most to the least sparse antigens, i.e. from the most to the least penetrating antibodies. This prevents the denser antigen from peripheral halo formation and from blocking antibody diffusion at the beginning. For example, diffused neurites labeling should be stained last.
7. Fluorescent probes with longer wavelengths offer a better signal-to-noise ratio, and it is preferable to match them with the least penetrating antibody. Near-infrared probes are strongly recommended if the microscope can support this spectrum.
8. Antigen density is the last determinant. Only sparse antigen abundance is appropriate for whole-brain immunostaining. A nuclear antigen is also preferred since neurite labeling obstructs antibody penetration. For dense antigens, e.g., NeuN or inhibitory interneurons, clearing with endogenous fluorescence-preserving protocols should be considered instead.

Box 4 **Tips for light-sheet microscopy and data analysis toolkit.**

3D microscopy of cleared brain ideally requires an objective lens with a long working distance (WD) and high numerical aperture (NA). WD refers to the distance between the first lens and the focal plane, which affects the field of view (FOV). NA refers to the angle of emitted light that is collected by the lens, which affects the resolution. Unfortunately, there is a trade-off in WD vs. NA. Typically, The NA is inversely proportional to WD. Therefore, high 3D resolution might eventually fail to encompass the whole brain. If the image exceeds the FOV or needs higher magnification, image tiling and stitching have to be done but that drastically lengthens the acquisition time and complicates the data handling.

Light-sheet microscopy (LSM) becomes the preferred method for imaging cleared brains owing to its fast and flexible imaging by uncoupling lateral and axial resolution via separate illumination and detection objectives. This raises the primary determinant for the users to choose their suitable LSM system: the horizontal focus. A sheet of light is always the thinnest and most focused at the center but progressively becomes thicker and loses focus toward its wrist. In order to achieve a uniform axial resolution, the LSM has to move the thinnest part of the light sheet across the whole FOV. The solution to horizontal focus is one of the main variations between different LSM systems, but also one of the key parameters to tune during image acquisition. Another major consideration in choosing the LSM system design is sample mounting, which in turn determines image orientation. For the purpose of brain-wide engram mapping, LaVision UltraMicroscope, which has been used in brain-wide activity mapping (Renier et al., 2016), or mesoSPIM, which employs axially scanned light-sheet microscopy (ASLM) for horizontal focusing (Voigt et al., 2019), are recommended to accommodate the need of multichannel imaging. For further LSM tutorials, we suggest the guide by Weiss et al. (2021). We also recommend the review of Daetwyler and Fiolka (2023) to get a glimpse of foreseeable breakthroughs in LSM development.

From the beginning, the big-data challenge of light-sheet imaging demands advanced hardware and software configuration from storage, reconstruction, to rendering (Reynaud et al., 2015). After meeting these prerequisites, data analysis involves mainly two stages: (1) cell detection and (2) atlas alignment. Whole-brain imaging is challenging to segment labelled cells owing to the highly heterogenous contrast from superficial to deep brain regions. This problem is worsened if the fluorescent label also fills up bright neurites. A standard pipeline for cell detection utilizes a sequence of filters to homogenize contrast and to create spherical objects, followed by intensity thresholding and morphological operation to define the cell. The standard pipeline is highly parametrical, so parameters are often estimated with a small training data of manually annotated images. Nowadays with the advancement of machine learning and artificial intelligence, non-parametrical pipelines, where the user provides ground truth data to train a model, are gaining in popularity. Detected cells must be allocated to brain regions to give regional statistics. The difficulty to register cleared brain images originates from non-uniform brain size deformation, which highly varies among clearing protocols. The standard pipeline for mouse brain atlas alignment matches the tissue autofluorescence channel with either Paxino's or Allen Brain Atlas by designing the transformation matrix. Common tools for cleared brain image analysis include BigStitcher, ClearMap, Imaris, and Arivis. A summary of the concept of a 3D image analysis pipeline is introduced here (Long et al., 2012).

Putting back into the context of brain-wide engram mapping, the final stage of data analysis is to disentangle functional networks and identify connectivity hubs. The standard computational framework involves interregional correlation matrix and graph theory analysis as depicted in Wheeler et al. (2013) or Silva et al. (2018), correlating cell count with behavioral readout as depicted in DeNardo et al. (2019), and *in silico* modeling as depicted in Vetere et al. (2017).

### What mechanistic aspects of the engram complex can be dissected with clearing?

**Figure 3:**
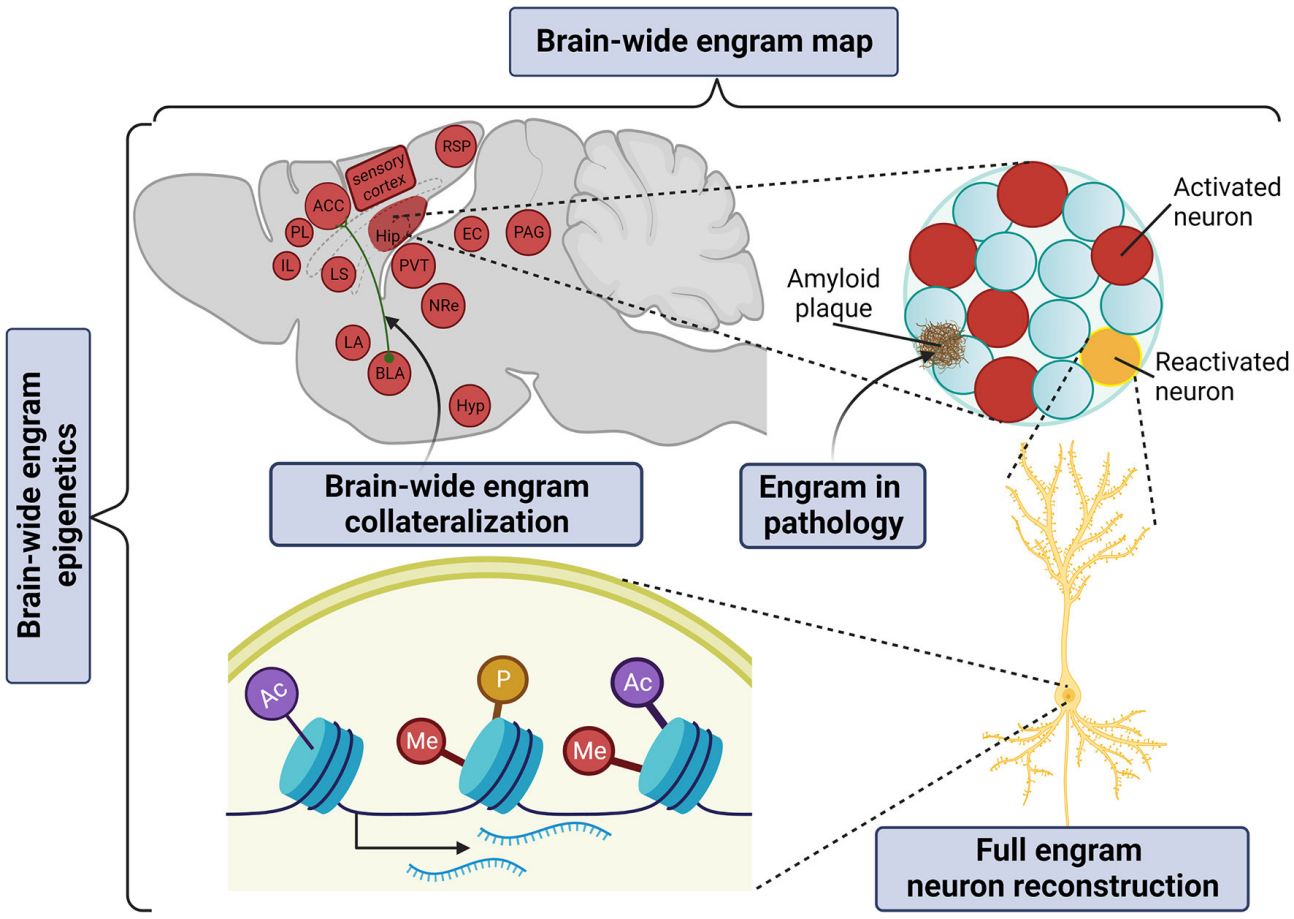
Application of tissue clearing to deconstruct the multilevel organization of the memory engram. Created with BioRender.com.

It is now widely accepted that the memory engram manifests itself as a multiscale organization of structural entities, spanning from circuits to molecules as potentially permanent and resilient substrates for information storage (Josselyn et al., 2015; Kyrke-Smith and Williams, 2018; Han et al., 2022; Ortega-de San Luis and Ryan, 2022). By achieving whole-brain multiplexed staining, optical clearing opens up the window to visualize these multilevel structures of the memory engram in just one composite snapshot (Figure 3).

#### Engram complex

The “engram complex” hypothesis suggests that among multiple engram cell ensembles of different brain regions, each supports a distinctive element of an experience but altogether express collectively as the memory. For example, for contextual memory, the hippocampal engram may represent context information (Sekeres et al., 2010; Park et al., 2016) or serve as an index of cortical engrams (Teyler and Rudy, 2007; Tanaka et al., 2014); the amygdalar engram may represent valence information (Liu et al., 2012; Redondo et al., 2014); prefrontal cortical engrams may serve as the top-down central hub of long-term memory storage (Frankland et al., 2004; Goshen et al., 2011; Dixsaut and Gräff, 2021), while the retrosplenial cortical engram may support the maturation and retrieval of remote memory (Todd et al., 2016; Lee et al., 2023). In addition, auditory and olfactory cortical engrams may represent and store sensory information (Fritz et al., 2005; Shakhawat et al., 2014; Abdou et al., 2018; Aqrabawi and Kim, 2020), thalamic nuclei may confer context discrimination and memory specificity (Ramanathan et al., 2018b; Troyner et al., 2018), the paraventricular thalamus may represent stress levels (Do-Monte et al., 2015; Penzo et al., 2015), the hypothalamic nucleus may represent threat levels for defensive responses under conditioned fear (Santos et al., 2008), while the periaqueductal gray may serve as the trigger of the freezing response (Kim et al., 1993; Tovote et al., 2015). Only in brain-wide manner can all brain areas of the “engram complex” participating in a given phenotype be visualized.

#### Engram circuit

Constructing a brain-wide engram-projectome map is of prime importance to address how engram reactivation is globally shaped by their structural innervations and functional recruitment. Speaking in non engram terms, a whole-brain collateralization map can already be generated with viral-genetic labeling in combination with whole-brain co-staining, which has first been illustrated by Ren et al. (2018), who addressed where serotoninergic collaterals project. Friedmann et al. (2020) subsequently developed Trailmap, a deep convolutional neural network pipeline to extract a mesoscale projectome. Trailmap successfully extracted activity-dependent thalamocortical axons in barrel cortex labeled by a conditional TRAP2-Cre × AAV-DIO-ChR2-mCherry. This approach laid the foundation of the brain-wide engram-collateralization map.

Nevertheless, a full reconstruction of engram cell remains challenging. The technical barrier of morphological reconstruction arises because thin axons of a diameter of ~100 nm often travel a long distance in the range of centimeters across multiple brain regions. Winnubst et al. (2019) established the contemporary standard of morphological reconstruction. By injecting a mixture of low-titer AAV Syn-iCre and a high-titer Cre-dependent fluorescent reporter, a very sparse set of approximately 20–30 cells per injection site could be labeled. The whole brain was optically cleared with AdipoClear, imaged with serial two-photon tomography at diffraction-limited resolution, and stitched serial stacks together into a single volume. In this way, more than 1,000 neurons in the neocortex, hippocampus, thalamus, and hypothalamus were reconstructed to unveil novel morphological subtypes and organizational principles.

#### Molecular engram

Despite the long history of molecular mechanisms underlying synaptic plasticity in learning and memory (Kandel, 2001), engram-specific molecular changes governing engram allocation, consolidation, and reactivation remain largely elusive. Since Francis Crick hypothesized that “memory might be coded in alterations to particular stretches of chromosomal DNA” more than 30 years ago (Crick, 1984), neuro-epigenetics has emerged as a fundamental mechanism underlying memory formation and storage. Epigenetic modifications, e.g., histone modifications and DNA methylation can react to short-lived neuronal activity and engrave the respective new information as long-lasting, yet reversible epigenomic programs. Marco et al. (2020) first elucidated fear engram cell-specific epigenetic modifications. This study used ArcCreERT2 × R26R-STOP-floxed eYFP to tag engram cells induced by auditory-cued fear conditioning and mapped engram cell-specific histone modifications, 3D genome architecture, and the transcriptional landscape for memory formation in the hippocampus. Together with Fernandez-Albert et al. (2019), who examined the epigenetic makeup of engram cells following novel object exploration, these studies found that learning triggers engram cells to switch from a permissive epigenetic signature to a maintenance transcriptomic signature that may facilitate long-term memory storage.

But how could an engram-specific epigenetic investigation be achieved at the whole-brain level? By intuition, it makes sense that epigenetic modifications as very dense markers are not amenable to be visualized in whole-brain imaging as nearly every single cell would be stained. Indeed to date, there is only one histone mark being examined with tissue clearing—the phosphorylation of H3S10 (H3S10p) to mark mitotic cells in E14 embryos using iDISCO (Renier et al., 2014). Interestingly, however, apart from its role as a cell proliferation marker, H3S10p has also been implicated in learning and memory as an activity-dependent epigenetic signal (Chwang et al., 2007; Koshibu et al., 2009, 2011; Wittmann et al., 2009; Gräff et al., 2012). For example, Chwang et al. (2006) used a dual-specific antibody to show that contextual fear conditioning triggers an increase of H3S10p concomitant with H3K14ac (H3S10pK14ac) in hippocampal area CA1, rapidly but transiently within 60 min. Thus, targeting activity-dependent combinatorial histone modifications with dual-specific antibodies might overcome the bottleneck of whole-brain immunostaining and thereby allow for brain-wide engram mapping at the epigenetic level. In addition, via RNA labeling (Kanatani et al., 2022) and reverse clearing, this could potentially extend into spatial transcriptomics to examine activity-dependent gene regulation.

#### Engrams in disease

With brain-wide engram mapping, it has even become possible to not only verify previous hypotheses about memory storage mechanisms in an engram-specific manner, but also to generate engram-centered, new hypotheses for neurological disorders. For example, Liebmann et al. (2016) demonstrated the first whole-brain triple co-staining of amyloid plaques, vasculature, and microglia in an Alzheimer's disease (AD) mouse model. This sparks the opportunity to spatially profile brain pathology with memory engrams in the context of memory loss. In addition, the possibility to co-stain glial cells together with engram cells might provide spatial information on how glia sculpts engram functionality. Interestingly, in another study, Roy et al. (2016) suggested that AD-related amnesia in AD mice might be due to a defect in memory retrieval rather than encoding. Nevertheless, mnemonic information acquired at encoding could be rescued by the direct activation of the DG engram. In future studies, it would be interesting to track how AD pathology progressively impairs engram accessibility or even ends with engram loss.

## Summary and outlook

Tissue clearing has revolutionized classical histology. This motivates the explosive proliferation of optical clearing protocols, but at the same time, it becomes challenging for researchers without any prior knowledge to select the best protocols regarding their experimental questions. The fact is that there is no one-size-fits-all method in tissue clearing. Even with basic knowledge of optical clearing, protocol optimization is still time-consuming mainly due to the lengthy protocol itself. In order to achieve the optimal 3D image quality, the best trade-off among diverse variables including tissue size change, deep signal intensity, tissue background autofluorescence, clearing formula complexity, imaging equipment, and analysis pipeline compatibility must be found. It is unlikely that there would be a simple plug-and-play solution without further optimization for the end user to harness the power of tissue clearing while specifically matching the needs of the biological question. At the same time, as all these technical complications demand multi-disciplinary knowledge to be overcome, tissue clearing has burgeoned into a stand-alone field of research. Therefore, tissue clearing will without a doubt continue to evolve in the foreseeable future. In turn, this will stimulate the popularity and advancement of brain-wide engram mapping to become technically less difficult and scientifically more robust. Eventually, the combined application of whole-brain engram tagging, whole-mount multiplexed staining, network computational pipelines, and engram-specific functional manipulations will expand our understanding of engram complex-specific functional connectomics and molecular repertoires in health and disease.

## Author contributions

KY and JG wrote the article. All authors contributed to the article and approved the submitted version.
